# Supplementary material for: Attosecond formation of charge-transfer-to-solvent states of aqueous ions probed using the core-hole-clock technique
Source: Nat Commun. 2024 Oct 16;15:8903. doi: 10.1038/s41467-024-52740-5 (PMC11480494; doi:10.1038/s41467-024-52740-5)
Supplement: Supplementary file 1 — Supplementary Information [file 41467_2024_52740_MOESM1_ESM.pdf]

Supplementary Information: Attosecond formation of charge-transfer-to-solvent states of aqueous ions probed using the core-hole-clock technique

E. Muchová*,[1] G. Gopakumar,[2] I. Unger,[2,3] G. Öhrwall,[4] D. Céolin,[5] F. Trinter,[6] I. Wilkinson,[7] E. Chatzigeorgiou,[2] P. Slavíček,[1] U. Hergenhahn,[6] B. Winter,[6] C. Caleman,[2,3] and O. Björneholm*[2]

[1]*Department of Physical Chemistry, University of Chemistry and Technology, Technická 5, 166 28 Prague, Czech Republic*

[2]*Department of Physics and Astronomy, Uppsala University, Box 516, SE-751 20 Uppsala, Sweden*

[3]*Center for Free-Electron Laser Science, DESY, Notkestr. 85, 22607 Hamburg, Germany*

[4]*MAX IV Laboratory, Lund University, Box 118, SE-22100 Lund, Sweden*

[5]*Synchrotron SOLEIL, L'Orme des Merisiers, Saint-Aubin, BP 48 91192 Gif-sur-Yvette Cedex, Paris, France*

[6]*Fritz-Haber-Institut der Max-Planck-Gesellschaft, Faradayweg 4-6, 14195 Berlin, Germany*

[7]*Institute for Electronic Structure Dynamics, Helmholtz-Zentrum Berlin für Materialien und Energie, 14109 Berlin, Germany*

muchovae@vscht.cz
olle.bjorneholm@physics.uu.se

Supplementary Note 1

Core-Excited-State Calculations The energy and intensity of the core-excited states have been modeled using various methods, here we focus on the time-dependent density functional theory (TDDFT) analysis with a restricted orbital space. The calculations and analyzes were performed for 50 structures from molecular dynamics (MD) simulations of $[M(H_2O)_6]^{n+}$ and $[M(H_2O)_{18}]^{n+}$ clusters representing the first and second solvation shells. The SRC2-R2 functional, specifically tailored to core excitations [43] with the cc-pVTZ basis set on water and the cc-pCVTZ basis set on the metal cation was used in all simulations. For all TDDFT calculations, the non-equilibrium solvation model was used as implemented in Q-Chem 6.0 [47].

The analysis of the excited-state wave functions was carried out using natural transition orbitals (NTOs), exciton analysis [48], charge-transfer numbers using the TheoDORE code [51], and the wave function analysis library (libwfa) [49, 50], to analyze the one-particle transition density matrix directly and to calculate the exciton and electron sizes as well as the charge-transfer numbers. We performed the analysis due to the complex nature of the excited states. Some of the properties of the excited states in the complex environment can be better understood using a two-body exciton wave function that describes the correlated motion of the hole and electron quasiparticles [48, 49, 50], rather than using the standard picture of molecular orbitals. The central quantity in this approach is the one-particle transition density matrix (1TDM, γ^{0I}) between the ground-state wave function, Φ^0, and the excited-state wave functions, Φ^I, which can also be interpreted as the electron-hole wave function $\chi_{\text{exc}}(r_e, r_h)$:

$$\chi_{\text{exc}}(r_e, r_h) = \gamma^{0I} = N \int \Phi^0(r_h, r_2, ..., r_N) \times \Phi^I(r_e, r_2, ..., r_N) dr_2, ..., dr_N, \tag{1}$$

where r_i denotes the spatial and spin coordinates of the i-th electron. The approach (discussed in detail in Refs. [49, 50]) allows the introduction of several practical quantities. The size of the exciton is often used. This is defined as the root-mean-square (RMS) separation between the instantaneous electron and hole positions (x_e, x_h):

$$d^2_{\text{exc}} = \left\langle |x_h - x_e|^2 \right\rangle = \frac{\langle \chi_{\text{exc}} | (x_h - x_e)^2 | \chi_{\text{exc}} \rangle}{\langle \chi_{\text{exc}} | \chi_{\text{exc}} \rangle}. \tag{2}$$

In the case of core-excited states of simple cations, both the hole (the 1s orbital) and the electron are centered on the cation. Therefore, the electron size (RMS size of the attachment density) is a suitable quantity to capture the difference between different cations, defined as:

$$\sigma_e = \sqrt{\langle x_e^2 \rangle - \langle x_e \rangle^2}. \tag{3}$$

a)

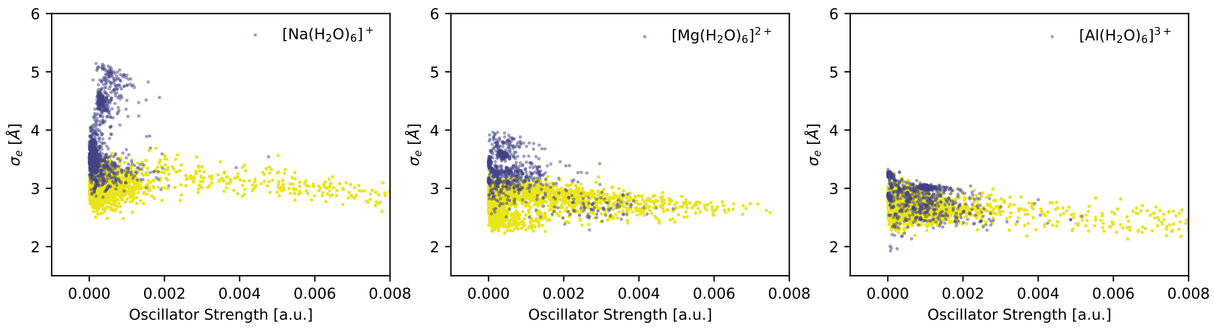

b)

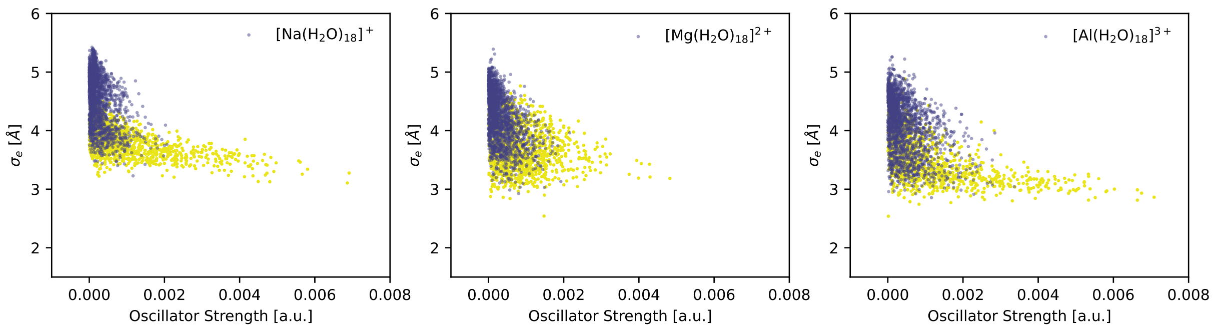

Supplementary Figure 1. Exciton analysis of the wave function (RMS electron size, σ_e) for 50 geometries of (a) $[M(H_2O)_6]^{n+}$ and (b) $[M(H_2O)_{18}]^{n+}$ at the SRC2-R2 level. The violet points correspond to excited states with excitation energies below the experimental ionization thresholds (see Supplementary Table 1), the yellow points correspond to the states above the respective thresholds.

In the case of organic molecules, the size of the exciton below $\approx$ 4 Å usually refers

to local excitations, while higher values indicate charge-transfer contributions. The values for solvated molecules with charge transfer to the solvent have not yet been studied, so no comparison is possible. From Supplementary Figure 2, we can conclude that in the case of the smaller $[M(H_2O)_6]^{n+}$ clusters, the electron sizes below the ionization threshold reach the highest values for $[Na(H_2O)_6]^{+}$ and the smallest values for $[Al(H_2O)_6]^{3+}$. In the case of the larger $[M(H_2O)_{18}]^{n+}$ clusters, the trend is similar but much less pronounced. However, the simple interpretation that the excitations of the $[Na(H_2O)_6]^{+}$ clusters below the ionization threshold indicate charge transfer to the water environment is premature due to the influence of the compactness of the solvation shell, which is the most compact for the $[Al(H_2O)_6]^{3+}$ and least compact for $[Na(H_2O)_6]^{+}$. However, we can conclude, that the "size" of the excited-state wave functions below the ionization threshold is largest for the Na^{+} cation and smallest for the Al^{3+} cation.

The charge transfer between the fragments, in our case between the metal cation and the surrounding water molecules, can be better described by different quantities. We can use the 1TDM γ^{0I} and decompose the excitation into different local (contributions on the metal cation) and charge-transfer contributions (contributions to the water molecules). For this purpose, we can define the so-called charge-transfer numbers:

$$\Omega_{AB} = \int_A dr_h \int_B dr_e |\gamma^{0I}(r_h, r_e)|^2, \tag{4}$$

where the hole is confined to a fragment A of the system and the electron to fragment B. In practical calculations, the actual charge-transfer-number analysis is a generalized population analysis:

$$\Omega_{AB} = \sum_{\mu \in A} \sum_{\nu \in B} (S^{1/2} D^{0I} S^{1/2})^2_{\mu\nu}, \tag{5}$$

where S is the overlap matrix and D^{0I} is the transition density matrix, defined as:

$$D^{0I}_{rs} = \left\langle \Phi^0 | E_{rs} | \Phi^I \right\rangle, \tag{6}$$

where r and s are two orbital indices and E_{rs} denotes the excitation from orbital s to orbital r. In TDDFT with restricted orbital space for core-excited states, the hole is always localized on the metal cation and instead of the Ω_{AB} matrix we get a vector, see Figure S3. Figure S3 shows the Ω_{AB} matrix for the first excited state of the $[Na(H_2O_6)]^+$ cluster and we can conclude that the state is predominantly locally excited (localized on the metal cation) and only a small part of the wave function is localized in the water environment (with the largest contribution on water number 4).

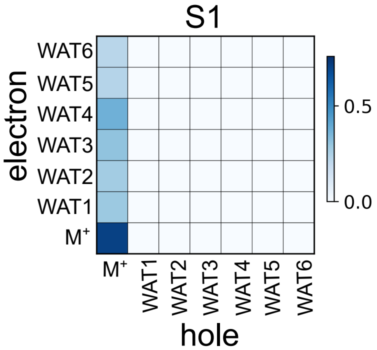

Supplementary Figure 2. Charge-transfer matrix for the first core-excited state (S1) for a selected structure of the $[Na(H_2O_6)]^+$ cluster. M^+ represents the metal cation fragment (Na^+), WAT1-WAT6 refers to the individual water molecules in the cluster. The intensity scale is on the right.

While Ω_{AB} matrix analysis can provide clear insights for complex molecules with charge-transfer-excited states, for the core-excited states we may be looking for more of a collective variable. We can define charge transfer CT by summing over all the off-diagonal elements, which gives us:

$$CT = \frac{1}{\sum_{A,B} \Omega_{AB}} \sum_{A,A \neq B} \Omega_{AB}. \tag{7}$$

This corresponds to the total weight of configurations where the initial and final orbitals are situated on different fragments. In this way, CT is 1 for all fully charge-separated states and 0 for locally excited states. In our case, we are interested in the weight of the configurations located on the same fragment, on the core-excited metal cation. We then calculate this weight as $1 - CT$. We can use the $1 - CT$ value and "project" it onto the oscillator strengths of individual excited states to quantify the portion of the excited-

state intensity that is due to the localized states on the metal cation, see Figure 1 in the manuscript.

a)

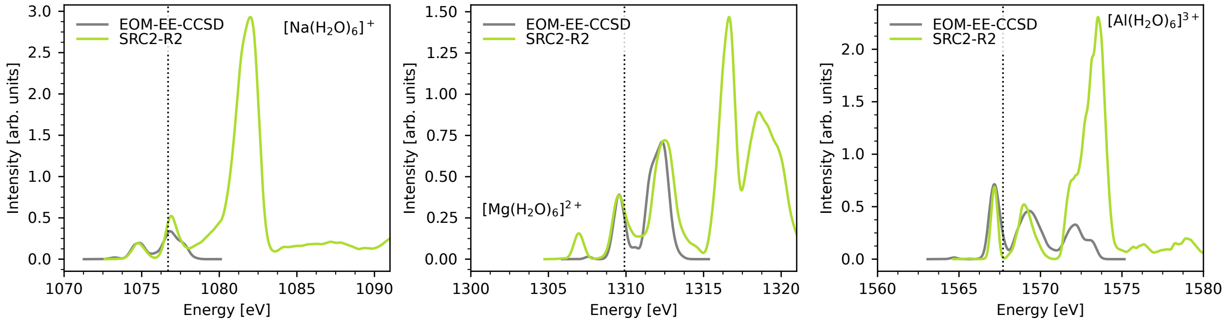

b)

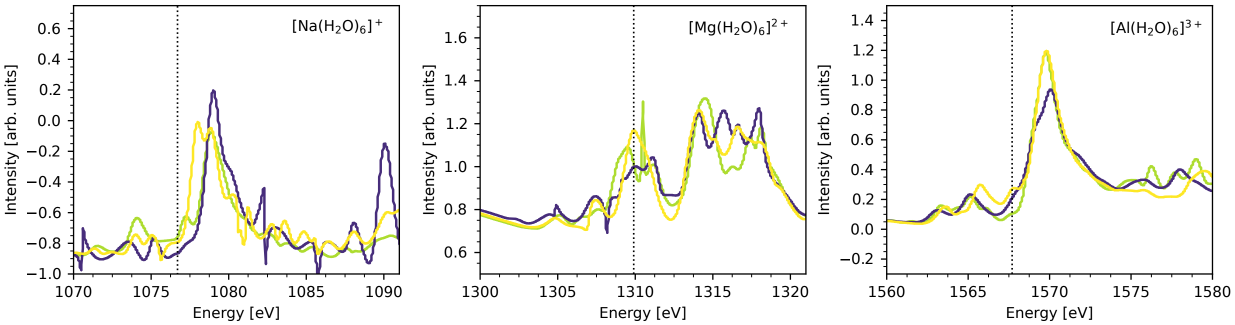

Supplementary Figure 3. Comparison between the onset of the spectra calculated at the (a) CVS-EOM-EE-CCSD level for a set of 50 structures and the SRC2-R2 level for a set of 200 structures for $[M(H_2O)_6]^{n+}$. The spectra at the SRC2-R2 level were shifted to match the CVS-EOM-EE-CCSD-level data (by 3.55, 2.70, and 4.40 eV, respectively). Each point of the spectra was broadened by phenomenological 0.2 eV. (b) Spectra calculated within the RT-TDDFT approach (three trajectories for each $[M(H_2O)_6]^{n+}$ cluster) at the SRC2-R2/cc-pVTZ level.

a)

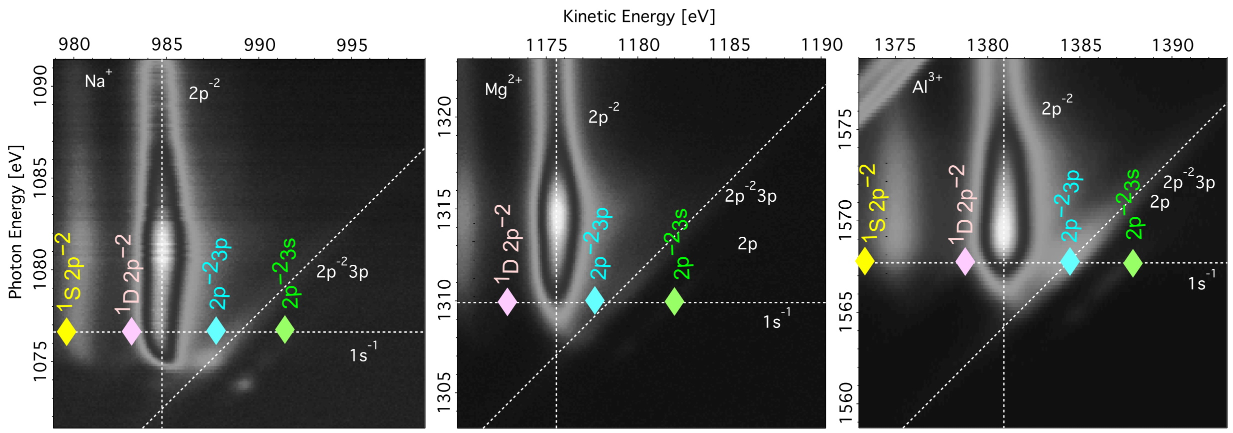

b)

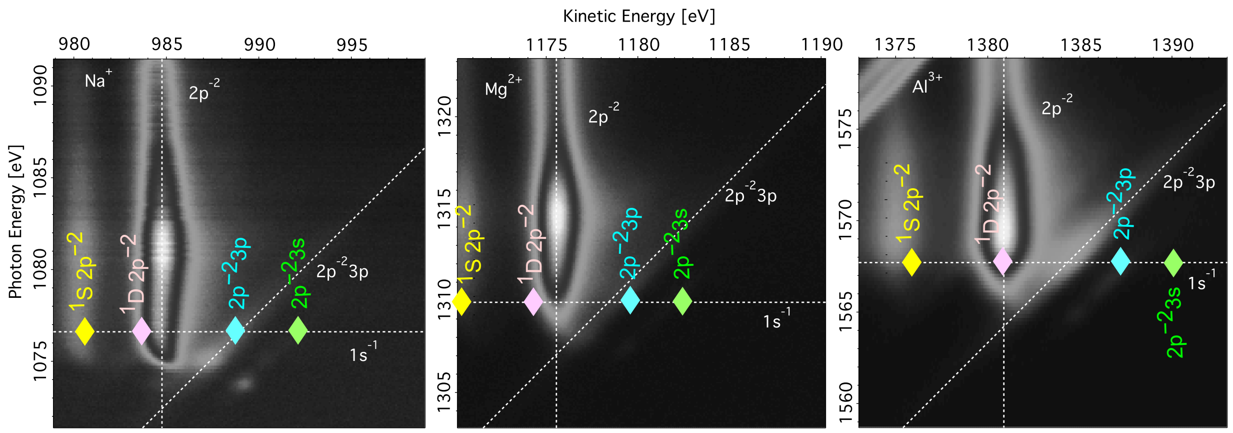

Supplementary Figure 4. 2D maps for the Na^{+}, Mg^{2+}, and Al^{3+} ions in water combined with visualization of the calculated energies at the ωB97X-D level, corresponding to the respective final states after AM decay. Energies for (a) $[M(H_2O)_6]^{n+}$ and (b) $[M(H_2O)_{18}]^{n+}$. The $^{1}S\ 2p^{-2}$ states for $[Mg(H_2O)_6]^{2+}$ are calculated to be below the experimental data onset at electron kinetic energies of 1170 eV.

EXPERIMENTAL DATA

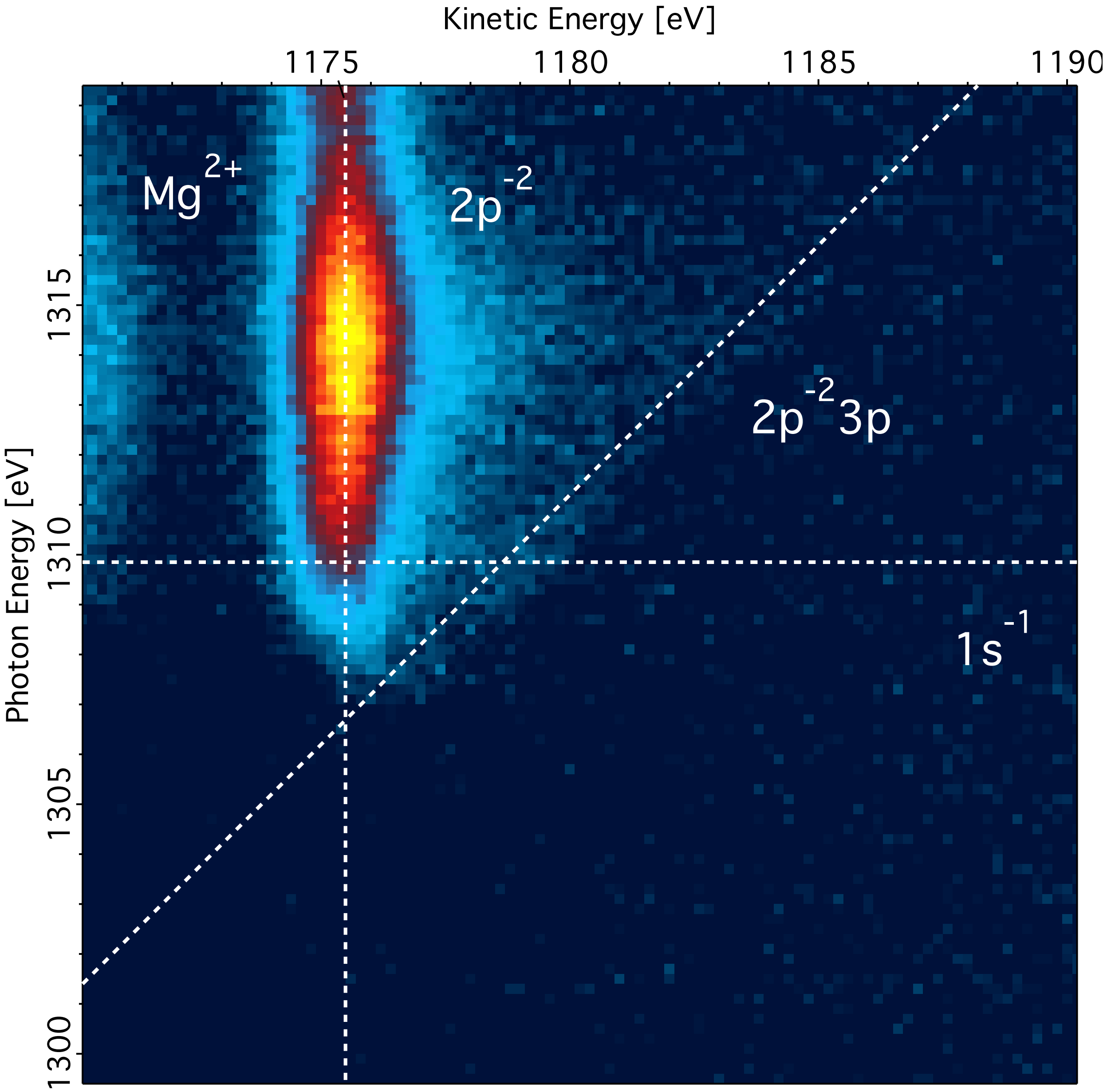

Supplementary Figure 5. 2D map showing the electron kinetic energy as a function of photon energy across the 1s ionization threshold for Mg^{2+} ions in water recorded at the HIPPIE beamline of MAX IV [40]. This 2D map is consistent with the corresponding 2D map recorded at beamline P04, shown in Figure 3 of the manuscript.

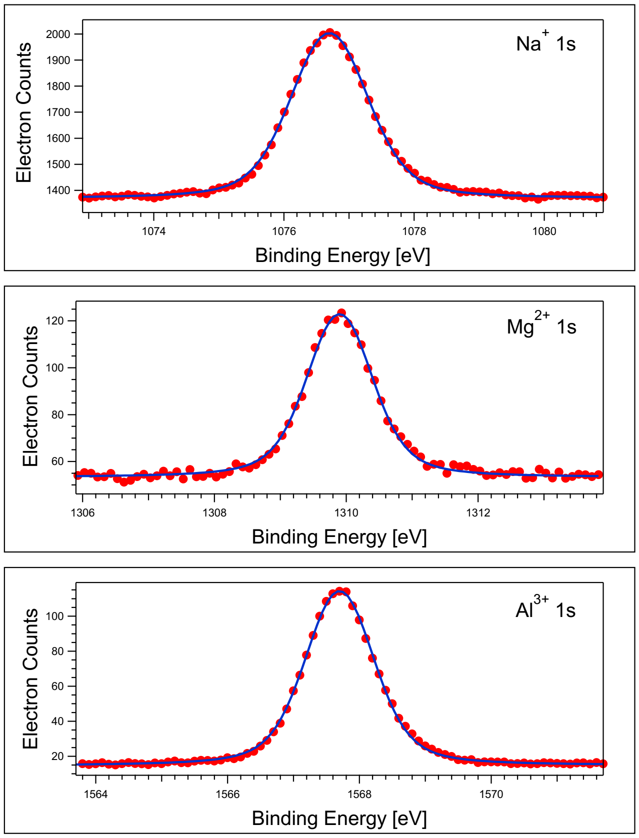

Supplementary Figure 6. Exemplary (a) 1 M Na^{+}_{aq}, (b) 1 M Mg^{2+}_{aq}, and (c) 2 M Al^{3+}_{aq} 1*s* photoemission spectra, as respectively recorded at photon energies of ∼1400, ∼1710, and ∼2168 eV at the P04 beamline using the *EASI* spectrometer system. (The Na spectrum is taken from Fig. S3 of Ref. [36].) Voigt-profile (blue) fits to the data (red circles) are shown in the middle of each figure panel, with the fit residuals shown at the top of each panel. Total peak full-width-half-maxima (FWHMs) of (a) 1.154 ± 0.008 eV, (b) 1.196 ± 0.0015 eV, and (c) 1.247 ± 0.007 eV were extracted from the Voigt-profile fits together with corresponding Lorentzian-fit-component FWHMs (Γs) of 0.45 ± 0.04 eV, 0.46 ± 0.06 eV, and 0.44 ± 0.03 eV. More generally, average Mg^{2+}_{aq} and Al^{3+}_{aq} Γ values of 0.47 ± 0.13 eV and 0.57 ± 0.32 eV have been determined from our aqueous-phase data sets recorded at photon energies between 1460-1710 eV and 1668-2568 eV, respectively, where the uncertainty ranges correspond to the standard deviation of the results extracted from the available data. As only a single Na^{+}_{aq} data set, shown in panel (a), was recorded, this prevented the assessment of the reproducibility and uncertainty associated with the determined Na^{+}_{aq} Γ value. However, as referred to in the main body of the text, the uncertainty ranges associated with the Γ values extracted from the Mg^{2+}_{aq} and Al^{3+}_{aq} M 1*s* photoemission data overlap with, and are correspondingly found to be in good agreement with, those of the metallic-sample, experimental data tabulated in Ref. [44].

Energy of state/decay	exp. Al^{3+}_{aq}	$[Al(H_2O)_6]^{3+}$	$[Al(H_2O)_{18}]^{3+}$
E($1s^{-1}$)	1567.7		
KE(KLL Auger–Meitner) ^{1}D	1380.9	1378.2 ± 0.3	1380.9 ± 0.9
KE(KLL Auger–Meitner) ^{1}S	≈1375	1373.3 ± 0.3	1375.9 ± 0.9
KE(KLL Auger–Meitner) ^{3}P	-	1383.5 ± 0.3	1386.2 ± 0.9
KE($2p^{-2}3p$)	≈1384.3	1384.7*	1387.8*
KE($2p^{-2}3s$)	≈1387.5	1387.7 ± 0.3	1390.7 ± 0.8
Energy of state/decay	exp. Mg^{2+}_{aq}	$[Mg(H_2O)_6]^{2+}$	$[Mg(H_2O)_{18}]^{2+}$
E($1s^{-1}$)	1309.9		
KE(KLL Auger–Meitner) ^{1}D	1175.6	1172.6 ± 0.5	1174.2 ± 0.5
KE(KLL Auger–Meitner) ^{1}S	≈1170	1168.5 ± 0.5	1170.1 ± 0.5
KE(KLL Auger–Meitner) ^{3}P	-	1176.9 ± 0.5	1178.5 ± 0.5
KE($2p^{-2}3p$)	≈1178.6	1177.2*	1179.5*
KE($2p^{-2}3s$)	≈1181.8	1181.4 ± 0.2	1182.2 ± 0.2
Energy of state/decay	exp. Na^{2+}_{aq}	$[Na(H_2O)_6]^{2+}$	$[Na(H_2O)_{18}]^{+}$
E($1s^{-1}$)	1076.7		
KE(KLL Auger–Meitner) ^{1}D	984.6	983.0 ± 0.9	983.8 ± 0.9
KE(KLL Auger–Meitner) ^{1}S	≈980	979.8 ± 0.9	980.6 ± 0.9
KE(KLL Auger–Meitner) ^{3}P	-	986.6 ± 0.9	987.4 ± 0.9
KE($2p^{-2}3p$)	≈988.7	987.5*	988.5*
KE($2p^{-2}3s$)	≈992.0	990.9 ± 0.4	991.5 ± 0.5

Supplementary Table 1. Average energies (E) and energy differences of the various charged electronic states and associated electron kinetic energies (KE), all values are given in eV. Where possible, experimental values are presented in the exp. M^{n+}_{aq} column, with the simulated results for the smaller and larger clusters shown in the $[M(H_2O)_6]^{n+}$ and $[M(H_2O)_{18}]^{n+}$ columns, respectively. The energy differences between the ^{3}P, ^{1}D, and ^{1}S states produced following Auger–Meitner decay were taken from calculations at the MRCI/aug-cc-pVTZ level, performed for cations in the gas phase. The energy differences between the ^{3}P and ^{1}D states and between the ^{3}P and ^{1}S states are for Na^+ 3.6 and 6.8 eV, for Mg^{2+} 4.3 and 8.4 eV, and for Al^{3+} 5.3 and 10.2 eV. *Only a limited number of data was obtained due to poor convergence of the MOM calculations.

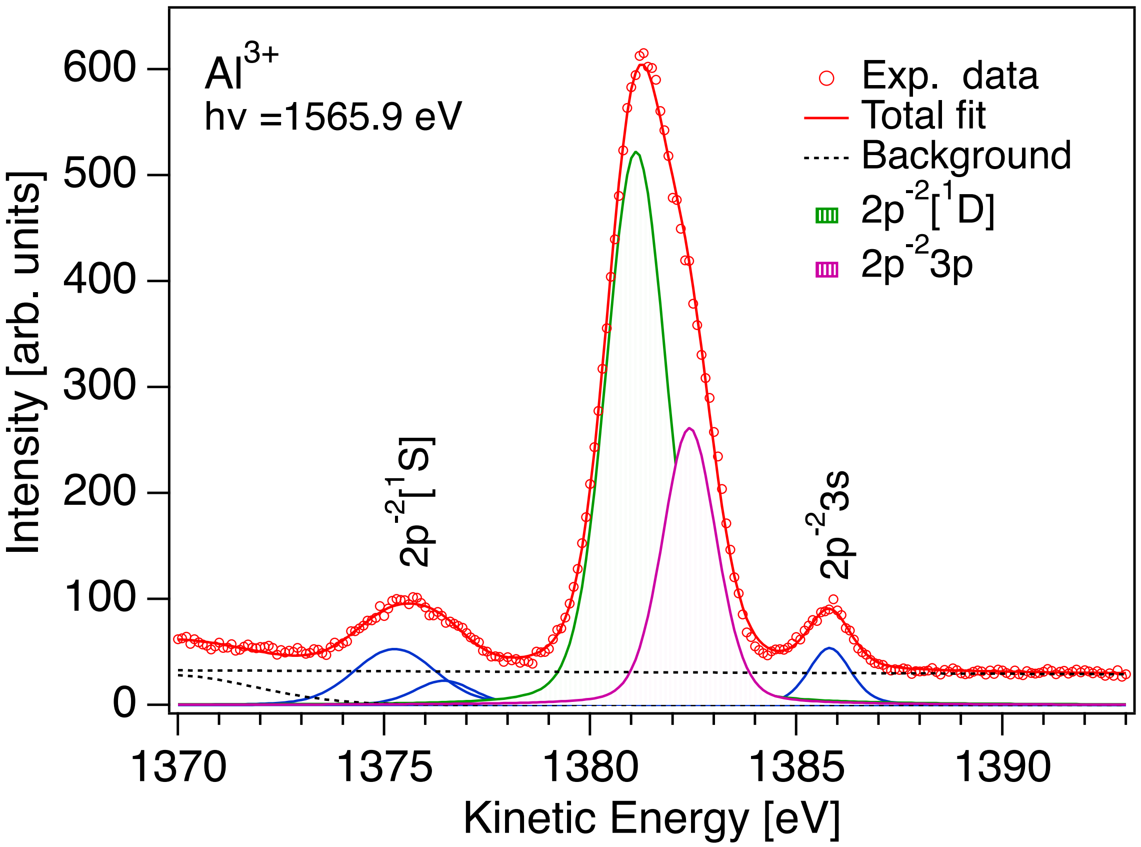

Supplementary Figure 7. Exemplary fit to the data derived from Figure 3 in the main text, for Al^{3+}_{aq} at the photon energy 1565.9 eV (slightly below the ionization threshold), as performed using the fitting procedure discussed in the main text. At this photon energy, the regular Auger-Meitner $2p^{-2}[^1S,^1D]$ features and the resonant $2p^{-2}[^1S,^1D]3p$ features overlap substantially. In addition to these states, a feature attributed to $2p^{-2}[^1D]3s$ can be seen at ≈1386 eV. In the analysis, only the states relating to the more intense $2p^{-2}[^1D]$ feature were used, and in Supplementary Table 2 the energies and intensities (peak areas) of the $2p^{-2}[^1D]$ and $2p^{-2}$ $[^1D]3p$ features obtained from the fits are exclusively presented.

Al_{aq}^{3+}	$2p^{-2}$	$2p^{-2}$	$2p^{-2}3p$	$2p^{-2}3p$	$2p^{-2}3p$	$2p^{-2}3p$	
Photon energy	KE	Int.	KE	Tot. Int.	Non-res. Int.	Res. Int.	Deloc. time
1565.9	1381.1	10480	1382.3	4714	228	4485	646
1566.9	1380.8	29280	1383.3	9618	228	4390	484
1567.9	1380.7	64970	1384.3	14010	228	13782	320
1568.9	1380.7	96581	1385.2	12410	228	12181	190
1569.9	1380.7	96899	1386.0	8155	228	7927	124
1570.9	1380.6	91006	1386.8	5140	228	4912	81
Mg_{aq}^{2+}	$2p^{-2}$	$2p^{-2}$	$2p^{-2}3p$	$2p^{-2}3p$	$2p^{-2}3p$	$2p^{-2}3p$	
Photon energy	KE	Int.	KE	Tot. Int.	Non-res. Int.	Res. Int.	Deloc. time
1308.95	1175.7	13865	1177.4	4569	82	4486	498
1309.95	1175.5	34517	1178.5	4140	82	4058	181
1310.95	1175.4	64185	1179.4	4148	82	4065	98
1311.95	1175.4	86898	1180.3	3495	82	3413	60
1312.95	1175.4	100577	1181.3	2269	82	2187	33
1313.95	1175.4	114792	1182.1	1687	82	1605	22
Na_{aq}^{+}	$2p^{-2}$	$2p^{-2}$	$2p^{-2}3p$	$2p^{-2}3p$	$2p^{-2}3p$	$2p^{-2}3p$	
Photon energy	KE	Int.	KE	Tot. Int.	Non-res. Int.	Res. Int.	Deloc. time
1076.24	984.6	29941	988.3	2732	289	2443	191
1077.24	984.5	33192	989.2	1619	289	1330	94
1078.24	984.6	34850	990.2	953	289	664	45
1079.24	984.6	37362	991.2	561	289	272	17
1080.24	984.6	40465	992.2	429	289	140	8
1081.24	984.6	41827	993.0	291	289	2	0

Supplementary Table 2. Kinetic energies (KE) and intensities (Int.) of the peaks used to estimate the electron delocalization times. The non-resonant intensity is estimated as the average of the $2p^{-2}3p$ peak area for the five lowest photon energies in the maps presented in Figure 3 in the main text. The energies are given in eV, intensities in arbitrary units, and delocalization times in as.
